# Data lake-driven analytics identify nocturnal non-dipping of heart rate as predictor of unfavorable stroke outcome at discharge

**DOI:** 10.1007/s00415-023-11718-x

**Published:** 2023-04-20

**Authors:** Alexander Nelde, Markus G. Klammer, Christian H. Nolte, Helena Stengl, Michael Krämer, Regina von Rennenberg, Andreas Meisel, Franziska Scheibe, Matthias Endres, Jan F. Scheitz, Christian Meisel

**Affiliations:** 1 Department of Neurology with Experimental Neurology, Charité-Universitätsmedizin Berlin, Bonhoefferweg 3, 10117 Berlin, Germany; 2 Center for Stroke Research Berlin, Berlin, Germany; 3 Berlin Institute of Health, Berlin, Germany; 4 German Center for Cardiovascular Research (DZHK), Partner Site Berlin, Berlin, Germany; 5 NeuroCure Cluster of Excellence, Charité-Universitätsmedizin Berlin, Berlin, Germany; 6 German Center for Neurodegenerative Diseases (DZNE), Partner Site Berlin, Berlin, Germany; 7 Bernstein Center for Computational Neuroscience, Berlin, Germany; 8 NeuroCure Clinical Research Center, Berlin, Germany

**Keywords:** Heart rate, Stroke outcome prediction, Data warehouse, Heart rate variability, Machine learning, Nocturnal non-dipping

## Abstract

**Background:**

Post-stroke heart rate (HR) and heart rate variability (HRV) changes have been proposed as outcome predictors after stroke. We used data lake-enabled continuous electrocardiograms to assess post-stroke HR and HRV, and to determine the utility of HR and HRV to improve machine learning-based predictions of stroke outcome.

**Methods:**

In this observational cohort study, we included stroke patients admitted to two stroke units in Berlin, Germany, between October 2020 and December 2021 with final diagnosis of acute ischemic stroke or acute intracranial hemorrhage and collected continuous ECG data through data warehousing. We created circadian profiles of several continuously recorded ECG parameters including HR and HRV parameters. The pre-defined primary outcome was short-term unfavorable functional outcome after stroke indicated through modified Rankin Scale (mRS) score of > 2.

**Results:**

We included 625 stroke patients, 287 stroke patients remained after matching for age and National Institute of Health Stroke Scale (NIHSS; mean age 74.5 years, 45.6% female, 88.9% ischemic, median NIHSS 5). Both higher HR and nocturnal non-dipping of HR were associated with unfavorable functional outcome (*p* < 0.01). The examined HRV parameters were not associated with the outcome of interest. Nocturnal non-dipping of HR ranked highly in feature importance of various machine learning models.

**Conclusions:**

Our data suggest that a lack of circadian HR modulation, specifically nocturnal non-dipping, is associated with short-term unfavorable functional outcome after stroke, and that including HR into machine learning-based prediction models may lead to improved stroke outcome prediction.

**Supplementary Information:**

The online version contains supplementary material available at 10.1007/s00415-023-11718-x.

## Introduction

Stroke is a significant public health burden, leading to considerable disability and mortality worldwide [1]. Generally, 45.7% of stroke patients suffer from unfavorable long-term outcome after 3 years (modified Rankin Scale (mRS) > 2) and 5.4% die in-hospital [2]. The main culprits are non-modifiable factors such as stroke severity and pre-stroke disability, but also in-hospital complications like pneumonia, cardiac changes and increased intracranial pressure [2, 3]. Therefore, accurate prediction of stroke outcome upon admission is essential for identifying patients at risk for an unfavorable course and implementing appropriate interventions.

Cardiac metrics like heart rate (HR) and heart rate variability (HVR) have been described in several studies as a proposed clinical parameter for outcome prediction [4–7]. Changes of HR and HRV in stroke reflect dysregulation of the autonomic nervous system (ANS), which includes decreased parasympathetic activity as well as increased sympathetic activity. This dysregulation can even lead to myocardial injury in the absence of coronary artery disease (CAD) [3, 8, 9]. In stroke, HR and HRV changes can manifest specifically in their circadian rhythm [10]. Studies have shown that higher HR upon admission correlates with poor stroke outcome [11–16] and that reduced HRV is associated with higher mortality after stroke [17], suggesting that these metrics could be useful additional outcome predictors. However, current evidence is lacking on whether the addition of HR variables improves stroke outcome prediction models [12]. This shortcoming may have several reasons. First, the time points of outcome assessment have been highly variable in previous studies, ranging from 24 h to 7 years after stroke, which complicates a direct comparison. Second, the precise calculation method of HRV measures differs between studies and is susceptible to noise and artifacts, as well as pharmacological interventions (e. g. antiarrhythmics) [8, 18]. Third, most studies have relied on only a single, brief HR/HRV measurement, usually upon admission, which neglects the well-known circadian modulations of these metrics. There is evidence that the information contained in the entire circadian HR pattern might add additional information beyond single HR or short-term HRV measurements: in hypertensive individuals, a blunted HR dip during sleep was significantly associated with all-cause mortality after up to 14 years of follow-up showing a strong linear correlation independently from other variables [19]. At this stage, it is unclear whether this applies to stroke patients and assessment of circadian HRV changes is not routine.

With the advent of data lake capabilities, it is now possible to store and analyze comprehensive data from stroke patients, including continuous electrocardiography (ECG) over multiple days. The use of these monitoring data and their circadian dynamics can provide a deeper understanding of cardiac and autonomic nervous system (ANS) function in stroke and its utility to improve outcome prediction accuracy. Automated analysis of multi-day in-hospital data to detect atrial fibrillation (AF) and other arrhythmias has already found widespread use. Likewise, this study aims to explore the possibility of using the same approach to monitor HR and HRV over extended periods of time to enhance our understanding of cardiac and ANS function in stroke and improve outcome prediction accuracy to overcome the limitations of single time point measurements.

In this study, we aimed to investigate (1) whether circadian changes in HR and HRV in the first 48 h after stroke are associated with short-term stroke outcome using routinely collected ECG data accessible from a comprehensive data lake structure, and (2) whether existing machine learning (ML) models for stroke outcome prediction could be improved by adding HR as a variable.

## Materials and methods

### Dataset

All patients diagnosed with nontraumatic intracerebral hemorrhage (ICD-10: I61.-) or ischemic stroke (ICD-10: I63.-) admitted to one of two separate stroke units at Charité Universitätsmedizin Berlin, Berlin, Germany, in the period between October 2020 and December 2021 were initially selected. The two stroke units totalling 20 monitoring beds allowed data transfer and integration into the Data Warehouse Connect (DWC) system (Philips) for long-term storage of monitoring data. The Charité/BIH (Berlin Institute of Health) Health Data Lake (HDL), a Hadoop-based platform that allows storage of a multitude of clinical, epidemiological, laboratory, and monitoring data was used for further integration and analysis. Usage and analysis of the data were approved by the Institutional Review Board of Charité Universitätsmedizin Berlin. The ECG data were recorded with Philips MP30 and MP50 monitors and stored in the data lake. We collected all beat-to-beat intervals from heart beats marked as normal (i.e. sinus rhythm) for up to 48 h after admission. To ensure a meaningful comparison of the HRV measures between subjects, we excluded patients with implanted pacemakers (by excluding beats marked as paced), as well as patients with a known AF diagnosis. In both cases, HRV can be skewed significantly. Other cardiac diseases were not considered for patient exclusion. Beyond ECG measures, the data lake also included a comprehensive set of additional parameters from each patient, including laboratory values, clinical scores, and diagnoses.

### Calculation of heart rate and heart rate variability metrics

To calculate the time series-based metrics, the signal was first divided into 5-min segments and a median value was determined for each segment. For every patient, all available signal within the first 48 h post-admission was evaluated. Therefore, assuming the full 48 h of data were available, a patient will have 12 5-min segments per hour and day, resulting in a maximum of 24 5-min segments for each individual hour of day over the 2-day period post-admission. For a more robust estimate, hourly values were only considered if at least 3 of the possible 24 5-min segments were available for the respective hour. HR was calculated as the number of beats per minute. Five HRV measures were considered in this study: standard deviation of beat-to-beat intervals (SDNN), root mean square of successive differences (RMSSD), low frequency power (LF, 0.04–0.15 Hz), high frequency power (HF, 0.15–0.4 Hz) and LF/HF. To calculate frequency domain metrics (LF, HF), the data were resampled by a factor of 4 and interpolated using the UnivariateSpline function of Python’s scipy library. Subsequently, the power spectral density (PSD) was estimated by applying a periodogram function for LF and HF areas, respectively.

### Risk factors and predictive features used in machine learning and patient matching

Following previous work [2], an ensemble of risk factors was assessed to provide a benchmark for the ML-powered outcome prediction. Epidemiological factors included age (numeric) and sex (binary value). Other clinical parameters and scores included National Institute of Stroke Scale (NIHSS) at admission (numeric) which quantifies stroke severity, hypertension, diabetes, previous myocardial infarction, known CAD and stroke-associated pneumonia (all binary values). Following previous work [20], serial measurements of high-sensitivity cardiac troponin (assay characteristics: high-sensitivity troponin T, Roche Elecsys, Gen 5; 99th percentile upper reference limit = 14 ng/l; 10% coefficients of variation (CV) precision = 13 ng/l; limit of detection = 5 ng/l) were categorized into three groups: 0—no elevation (below 14 ng/l); 1—at least one elevated sample (above 14 ng/l); 2—acute elevation (one sample above 14 ng/l and a second sample with a 20% increase or decrease in respect to the first sample). Additionally, the glomerular filtration rate (GFR, numeric) was included. Bearing in mind previous work on functional dependence and mRS nomenclature [21], we dichotomized patient outcome into two groups. The first group consisted of patients considered able to live independently, defined by mRS 0–2 upon discharge. The second group included all patients with varying degrees of dependency (including death), i.e., mRS 3–6 upon discharge. From here on out, the two groups are referred to as patients with good vs. unfavorable outcome, respectively. As expected, the two patient groups displayed a high imbalance in age and NIHSS distributions. Given that HR and HRV are known to be age- and NIHSS-dependent, we matched patients according to age and NIHSS to avoid bias [22]. Patients were matched with a propensity score matching (PSM) algorithm (Python library PsmPy) using the k-nearest neighbors method without match replacement and a caliper of 0.2.

### Machine learning

We analyzed two ML models with varying degrees of complexity to assess the relevance of HR measures to improve stroke outcome prediction and to validate the robustness of our approach. Specifically, we employed supervised ML to predict the outcome in two classes, good and unfavorable outcome, based on mRS values at discharge. We assessed algorithm performance using a nested cross-validation approach where the dataset was randomly split 4:1 into training and testing sets in a way that the folds preserve the percentage of samples for each class. In a nested fivefold cross-validation, the model’s hyperparameters were tuned and an average model performance calculated over the five folds. This nested cross-validation allowed us to estimate an unbiased performance of the model’s ability to generalize on held-out test sets that were not part of the hyperparameter tuning. This process was repeated 50 times in random shuffles. The hyperparameter ranges used for tuning are displayed in Suppl. Table 1. Data features were normalized by removing the mean and scaling to unit variance. The models included Logistic Regression and CatBoost. CatBoost is a non-linear model based on gradient boosting, which attempts to solve for categorical features using a permutation-driven algorithm. Compared to the previous models, gradient boosting is a more advanced framework for solving regression and classification tasks. For classification, the prediction model is built as an ensemble of weak classification models (classifiers), typically in the form of decision trees. All weak classifiers are then integrated into one cumulative model, that performs better than any of its individual classifiers. In this study, gradient boosting was implemented using the CatBoost library developed by Yandex in 2017.

### Machine learning interpretability

In medical applications, the interpretability of ML frameworks is crucial to improve the understanding and acceptance of ML results. In linear models, the models’ coefficients are commonly interpreted as the global feature importance of the respective variables. As such, they may refer to the usefulness of each feature for the prediction. This holds true, if the variables are not exhibiting collinearity, as is the case in this study. For non-linear models a different approach is required to extract a similar relative metric. One recent strategy was derived from game theory and is known as Shapley Additive Explanations. The idea is to compare how well a model performs with and without a feature for every combination of features, therefore calculating a local feature importance for every permutation, as opposed to the global importance that is derived from coefficients and constitutes a single ranking of all features for the model (Table 1). Nevertheless, a global measure can be gained by accumulating local features for every data point. For comparability across different models, both feature importance metrics were normalized (i.e., scaled to unit norm).

**Table 1:** Values used for tuning hyperparameters in machine learning models

Model	Hyperparameter	Range
Logistic regression	Inverse of regularization strength	0.1, 0.12, 0.15, 0.18, 0.21, 0.26, 0.31, 0.37, 0.45, 0.54, 0.66, 0.79, 0.95, 1.15, 1.39, 1.68, 2.02, 2.44, 2.95, 3.56, 4.29, 5.18, 6.25, 7.54, 9.1, 10.9, 13.3, 16.0, 19.3, 23.3, 28.1, 33.9, 40.9, 49.4, 59.6, 72.0, 86.9, 105.0, 126.0, 153.0, 184.0, 222.0, 268.0, 324.0, 391.0, 471.0, 569.0, 687.0, 829.0, 1000.0
CatBoost	Tree depth	2, 4, 6
	Learning rate	numpy.linspace(0.03, 0.3, 20)
	Bagging temperature	numpy.linspace(0.6, 1, 3)
	L2 leaf regularisation	3, 10, 100, 500
	Leaf estimation iterations	1, 2

### Performance assessment and statistical evaluation

ML model performance was assessed on the held-out test sets by calculating the area under the receiver-operator-characteristics (AUC). We reported the mean over the number of shuffles. For HR and HRV measures, statistical independence between groups for each hour of the day was tested by Mann–Whitney-*U* test with additional Bonferroni correction for multiple comparison (24 individual hours). A two-sample *t* test was used to compare ML models with and without HR metrics.

## Results

**Fig. 1:**
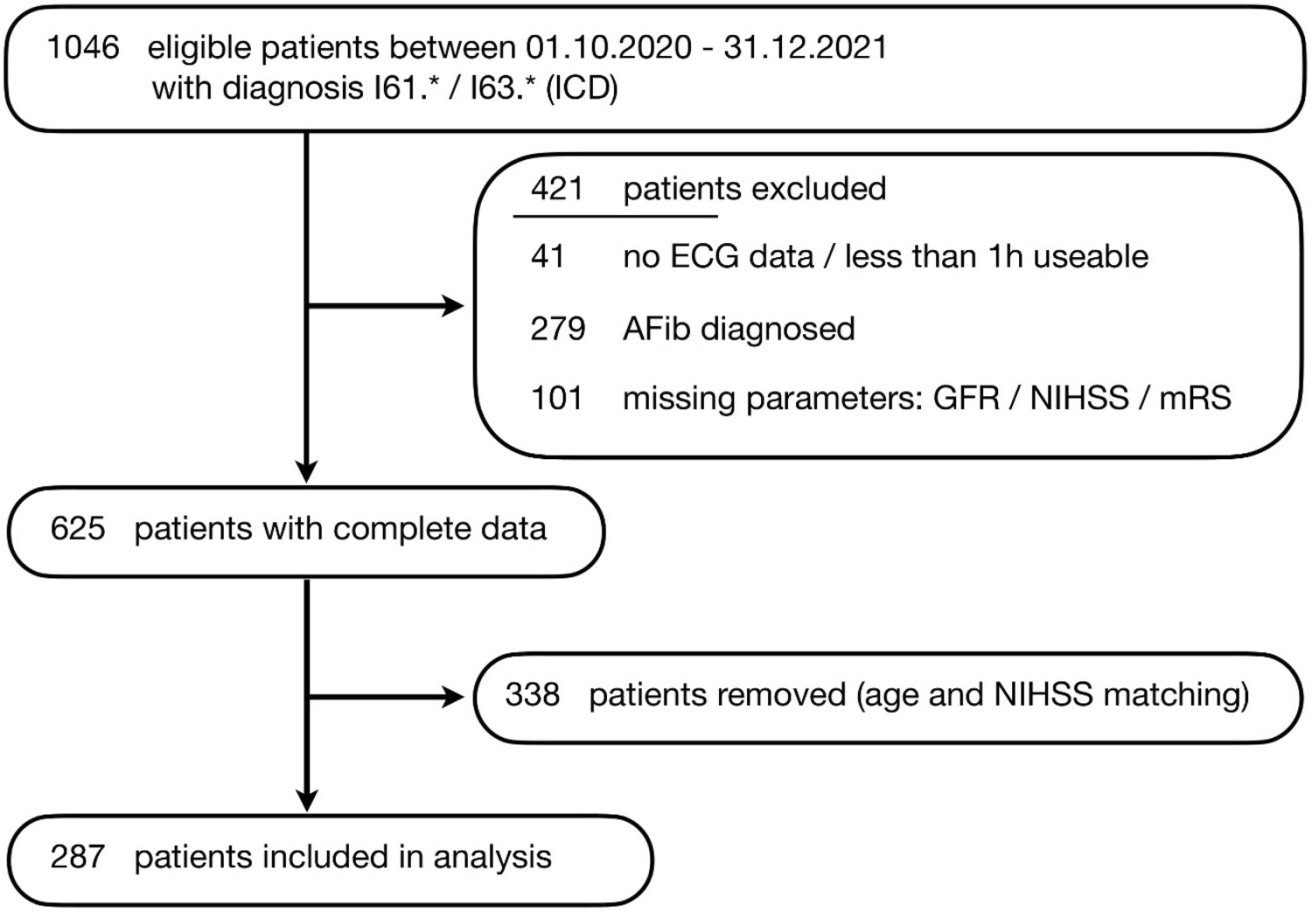
Flow-chart of patient selection

We identified 1046 eligible patients across two Charité stroke units admitted between October 2020 and December 2021 matching our diagnosis criteria (Fig. 1). From the initial selection, 421 patients were excluded due to insufficient numbers of normal heart beats labeled for accurate determination of HR/HRV metrics (*n* = 41), missing data points such as NIHSS, mRS or laboratory parameters like GFR (*n* = 101) and patients who were diagnosed with AF either during the current hospital stay or in the past (*n* = 279). We explicitly excluded patients with AF due to the potential impact on HRV metrics, but later checked consistency of our main findings for all patients, including patients with AF. Because HRV metrics and ML performance are known to be highly dependent on age and NIHSS, the remaining 625 patients were matched according to age and NIHSS between groups with good vs. unfavorable outcome, reducing the number of patients considered in the subsequent analysis to 287 (255 with ischemic stroke, 32 with intracranial hemorrhage; Fig. 1). The main patient characteristics are displayed in Table 2.

**Table 2:** Characteristics of patients with good (mRS 0–2) and unfavorable outcome (mRS 3–6)

Parameters	Total	Good	Unfavorable	*p* value
Number of patients	287	121	166	
Epidemiological				
Mean age (years)	74.5	71.8	76.6	< 0.01
Sex (female/male) [%]	131/156 [45.6/54.4]	47/74 [35.6/47.4]	84/82 [64.4/52.6]	< 0.05
Clinical				
NIHSS (median)	5.0	5.0	6.0	< 0.01
Pneumonia [%]	15 [5.2]	1 [0.8]	14 [8.4]	< 0.01
Hemorrhagic stroke [%]	32 [11.1]	7 [5.8]	25 [15.1]	< 0.05
Ischemic stroke [%]	255 [88.9]	114 [94.2]	141 [84.9]	< 0.05
IVT^a^ [%]	71 [24.5]	39 [32.2]	32 [23.5]	< 0.05
EVT^a^ [%]	39 [13.6]	22 [18.18]	17 [10.24]	0.05
Pre-existing conditions:				
Diabetes [%]	83 [28.9]	34 [28.1]	49 [29.5]	0.79
Hypertension [%]	240 [83.6]	89 [73.6]	151 [91.0]	< 0.01
Myocardial infarction [%]	3 [1.0]	1 [0.8]	2 [1.2]	0.76
Coronary artery disease [%]	26 [9.1]	12 [9.9]	14 [8.4]	0.67
Laboratory values				
Troponin (0/1/2)^b^ [%]	104/106/77 [36.2/36.9/26.8]	53/43/25 [43.8/35.5/20.7]	51/63/52 [30.7/38.0/31.3]	< 0.05
Mean GFR [ml/min]	74.1	74.7	73.7	0.64
HRV measures (mean)				
Heart rate [1/min]	71.3	68.3	73.5	< 0.01
SDNN [ms]	48.4	46.6	49.7	0.44
RMSSD [ms]	43.2	39.3	46.0	0.27
LF [ms^2^]	693.6	646.2	728.1	0.66
HF [ms^2^]	1056.9	849.2	1208.4	0.38
LF/HF [ms^2^]	2.3	2.5	2.1	0.09

**Fig. 2:**
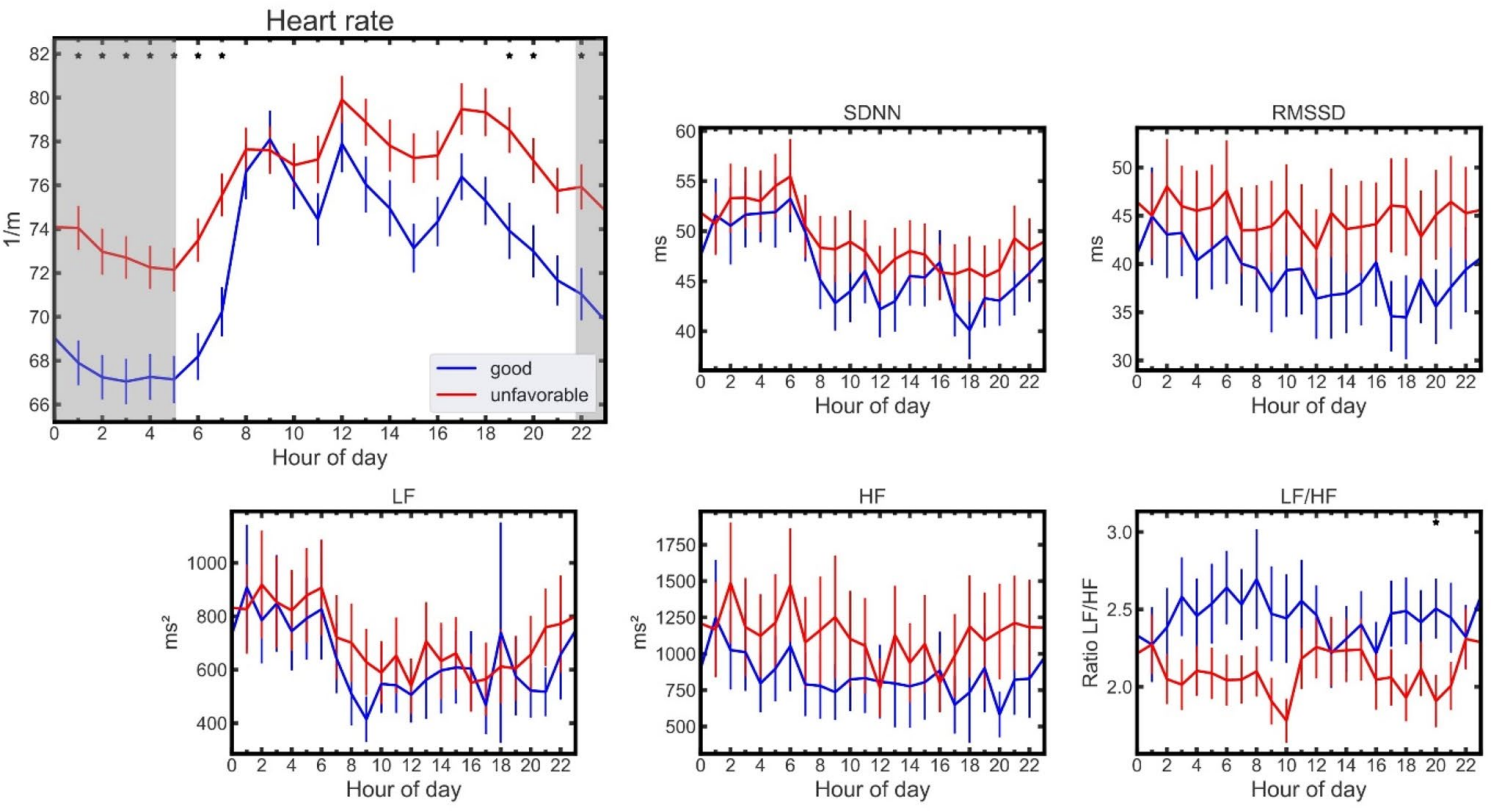
Circadian dynamics in HR and HRV of patients with good (blue) vs. unfavorable health state (red) after stroke. HR values are significantly lower in the good outcome group with a pronounced dip during the night (gray area). Values indicate means with standard deviation. *Indicates *p* < 0.05 for difference between respective individual hours, Mann–Whitney-*U*-Test, Bonferroni corrected

Heart rate variability (HRV) measures are reported as mean over the whole circadian cycle, with exception for the heart rate, which is averaged over the period 22:00–05:00 to capture the difference in circadian rhythms between groups (Fig. 2, gray area)

^a^Intravenous thrombolytic therapy (IVT), Endovascular treatment (EVT)

^b^Troponin coding, see “Methods”

### Circadian patterns of HR, HRV and their association with functional stroke outcome

Figure 2 shows HR and HRV metrics for patients with good (blue) and unfavorable outcome (red) after stroke. HR exhibited a significant difference between the two groups with good outcome generally being associated with a lower HR for several hours of the circadian time course. This difference was most pronounced during night hours. We quantified our findings by comparing both groups average HR decline during night hours (22:00–05:00) to a daytime average (10:00–17:00), yielding an average decline of 10.5 ± 2.1 % for patients with good outcome and an average decline of 6.3 ± 1.8 % for patients with an unfavorable outcome. Where the error is the standard error of the mean. Apart from 1 h in the LF/HF time course, no hourly differences were observed for HRV measures.

**Fig. 3:**
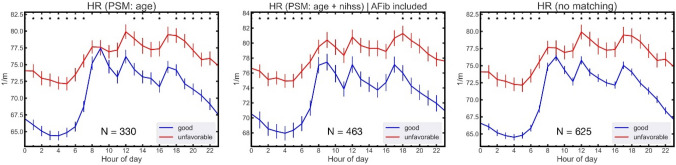
Nocturnal non-dipping of HR differentiates good from unfavorable stroke outcome at discharge. Nocturnal non-dipping of HR as a distinguishing feature of unfavorable outcome is robustly observed when patients are only matched for age (left), when patients with atrial fibrillation (AF) are included (middle) and when patients are *not* matched for either age or NIHSS (right). PSM: propensity score matching. Data displayed as mean with standard deviation. *Indicates *p* < 0.05 for difference between respective individual hours, Mann–Whitney-*U*-Test, Bonferroni corrected

Nocturnal non-dipping of HR differentiated good from unfavorable stroke outcome at discharge robustly, also when patients were only matched for age, when patients with atrial fibrillation (AF) were included, and when patients were not matched for either age or NIHSS (Fig. 3).

The robust observation of “dippers” vs. “non-dippers” may potentially serve to further categorizes these different patient groups. To provide a potential tool to differentiate the two groups, we quantified the HR dipping for the two different groups by comparing the average HR during nighttime (22:00–05:00) to the HR average during daytime (10:00–17:00). Patients with an unfavorable outcome exhibited a less pronounced dip in HR of 6.3 ± 1.8% on average. In contrast, patients with a good outcome exhibited a more pronounced dip in HR of 10.5 ± 2.1% on average when comparing day- to nighttime (10:00–17:00 vs. 22:00–05:00).

### Prediction of stroke outcome

**Fig. 4:**
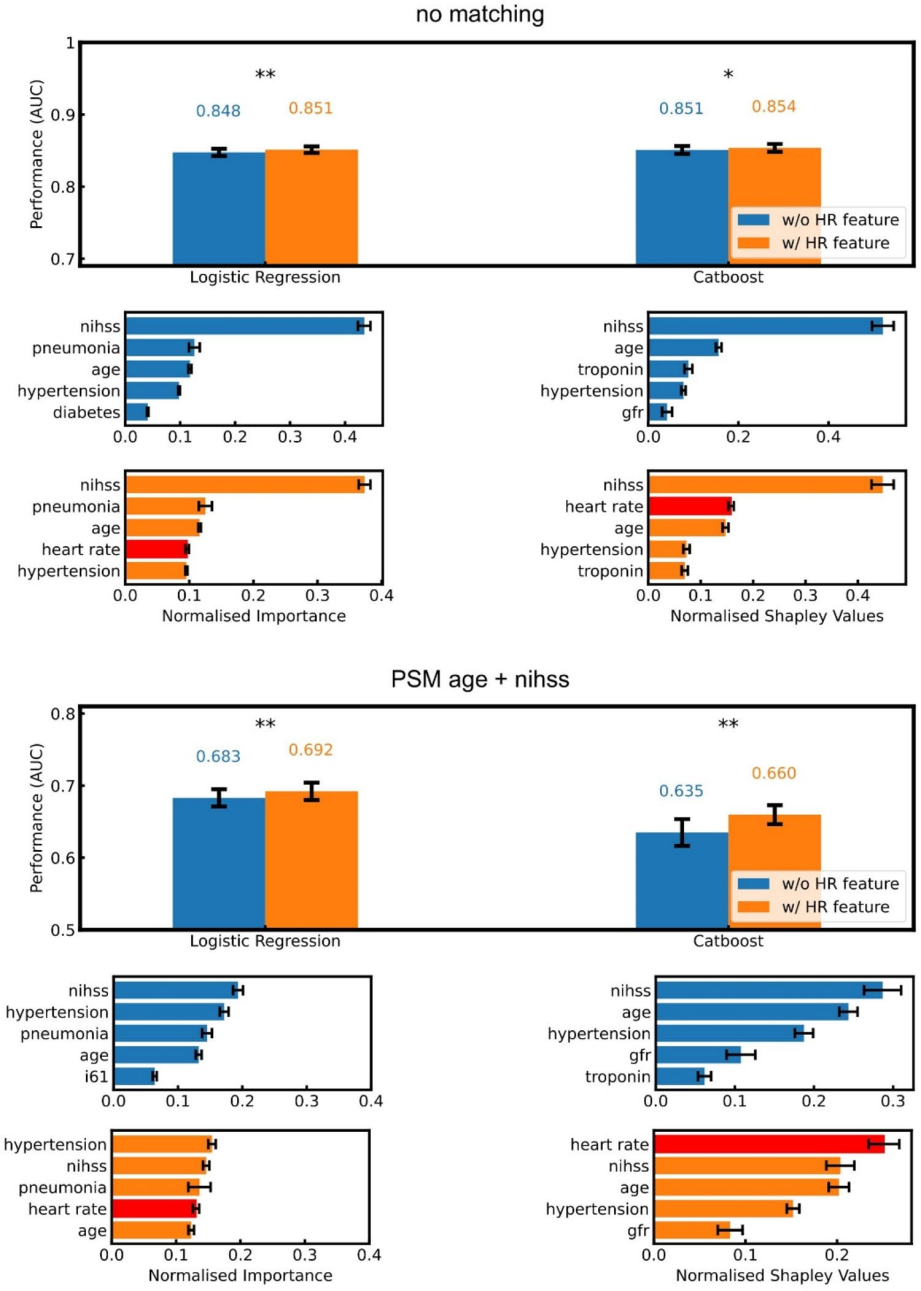
Nocturnal HR non-dipping improves stroke outcome prediction as an independent parameter. Top: results for non-matched patients. Bottom: results of patients matched by age and NIHSS. Comparison of prediction performance between feature groups with (orange) and without (blue) utilization of HR (beats per minute, bpm) for prediction and corresponding feature importance for the ML models. For every model type, HR feature importance ranks highly, implying an effective inclusion into the prediction decision. *AUC* area under the receiver-operator-characteristics, *PSM* propensity score matching. Data displayed as mean with standard deviation. *Indicates *p* < 0.05; **p < 0.001; standard two-sample location *t* test for 50 shuffles of nested cross-validation

Next, we investigated whether the difference in HR between groups with good vs. unfavorable outcome would improve ML-powered outcome prediction. To this end, we added nocturnal HR, i.e., the mean HR value from 10 pm to 5 am (Fig. 2, gray area), as an independent parameter to the listed clinical, epidemiological and laboratory factors in predicting stroke outcome (Table 2). After adding HR, the prediction performance increased for both models when applied to non-matched data (AUC 0.851 ± 0.005 logistic regression, AUC 0.854 ± 0.005 CatBoost) and when applied to age- and NIHSS-matched data (AUC 0.692 ± 0.012 logistic regression, AUC 0.660 ± 0.013 CatBoost; Fig. 4). Analyzing individual feature importance consistently indicated a high relevance of the HR parameter in all models (Fig. 4, bottom). In conclusion, HR improved the overall performance of ML and was considered a useful additional feature in all models.

Finally, we performed a sub-analysis excluding patients diagnosed with intracranial hemorrhage. All results remained robust (Supplemental Table S1, Figures S1 and S2).

## Discussion

By analyzing continuous, data lake-enabled ECGs, we investigated whether good vs. unfavorable outcome after stroke was associated with different circadian HR/HRV profiles and whether these metrics could improve outcome prediction through ML. We found that patients with unfavorable functional short-term outcome showed an overall higher HR with a distinct lack of nocturnal HR-dipping, whereas HRV measures did not differ between the groups. We also found that incorporating HR into the ML models significantly improved the performance of the short-term outcome prediction models compared with models using conventional prognostic factors including stroke severity, age, sex, cardiac history [2, 23]. To the best of our knowledge, no other research to date has used continuous, circadian monitoring data of HR patterns for ML-based outcome prediction.

Our observation that higher HR is associated with unfavorable outcome is in line with several previous studies: resting HR in stroke patients is associated with overall mortality, acceleration of post-stroke cognitive decline and cardiovascular morbidity, especially in men, but not stroke recurrence [11, 13, 15]. Although comparability between these studies is limited, HR is still recognized as an independent risk factor for cardiovascular morbidity and mortality even after adjusting for other risk factors like dyslipidemia, and hypertension [15]. This is in line with our finding that including HR into ML models of stroke outcome prediction significantly improved their performance.

Numerous trials showed an independent relationship between HR and stroke, even after adjusting for the most common cardiovascular risk factors [24–26]. This relationship is likely due to the overactivity of the sympathetic nervous system, which seems to predominantly affect the first three days after stroke [15, 27, 28]. Additionally, approximately 50% of stroke patients suffer from insomnia [29]. It is hypothesized that ANS dysfunction is exacerbated during sleep and that sleep disruption after stroke leads to early clinical deterioration through ANS-mediated arrhythmias and blood pressure fluctuations [3]. Hence, we believe it is valid to view HR and its circadian modulation as a surrogate for post-stroke ANS dysfunction. Crucially though, our study also showed a distinct lack of nocturnal HR dipping in patients with unfavorable functional outcome after stroke; none of the studies discussed above differentiated between diurnal and nocturnal HR. It is unclear whether the associations reported by these studies are due to elevated HR in general or specifically due to the blunted HR dip. It should be mentioned that HR fluctuations could also be influenced by other conditions like post-stroke delirium: however, the retrospective analysis of delirium prevalence and its impact on outcome was not feasible within our cohort due to missing data.

Night-time HR dipping gained more interest around the turn of the millennium, when it was discovered that the physiological night-time dip is reduced in patients with CAD or diabetes [30]. A sizable study of 3957 patients showed a remarkably sturdy linear relationship between blunted HR dip and all-cause mortality in hypertensive patients [19]. Interestingly, awake HR was not associated with mortality, and night-time HR showed only a weak association, indicating that it might indeed be the degree of dipping that matters most. HR non-dipping has never been implicated in stroke outcome prediction, although it was suggested to increase the risk of cardiovascular events including stroke in patients treated for hypertension [31].

The impact of stroke on the ANS has been explored for almost 70 years now [32]: for example, HRV is severely decreased in acute ischemic stroke [33–35], likely due to autonomic dysregulation after stroke [36]. Some studies found a depression of parasympathetic activity in the acute and chronic stages of stroke, indicated by lower LF [10, 37], while others found that increased sympathetic activity could be the culprit of post-stroke autonomic dysregulation [38]. This dysregulation can lead to immunosuppression and has even been linked to post-stroke infections like pneumonia [39–41]. Other signs of post-stroke ANS imbalance include ECG alterations (QTc prolongation, AF, supraventricular and ventricular tachycardia) [42, 43], but also sleep disruption, altered baroreceptor reflex sensitivity, and reduced left ventricular ejection fraction [28, 44, 45].

HRV has been proposed as an independent marker to predict mortality in stroke patients [4, 46, 47]. However, an observational study investigating the predictive power of HRV regarding stroke outcome (n = 308) showed only an association between SDNN and mortality after 3 months; however, similarly to our study, no association of HRV and mRS after 3 months or 1 year could be identified [17].

We used continuous monitoring data to analyze HRV through a ML model. This might help explain, why our results differ from the bulk of the available literature: digesting large amounts of monitoring data will inherently yield different results from traditionally collected data. It is still common practice to calculate HRV measures using a 5–10 min ECG segment after manually eliminating artifacts and premature heart beats [48]. In our study, however, we harnessed the power of ML to analyze a much longer ECG segment.

There is evidence that post-stroke autonomic dysfunction and cardiac changes might be more pronounced in hemorrhagic than ischemic stroke, possibly due to higher stroke severity as well as time-course and extent of brain injury [3]. Therefore, we have accounted for differences in stroke severity by matching for baseline NIHSS scores. We also performed a sub-analysis excluding patients with intracerebral hemorrhage (ICD I61). The results did not change meaningfully.

### Limitations

Several limitations must be noticed in our study. First, the measurement of HRV in an acute clinical setting is challenging: HRV is influenced by numerous factors including intake of cardioactive drugs, time of day, and physical activity levels [49]. Second, the optimal method of HRV measurement is still a topic of debate. Initially, linear statistical methods were heavily employed for its calculation, but the last decade has seen a steady rise in non-linear approaches using methods from chaos theory and fractal analysis. Here, we analyzed a comprehensive, well-established set of HRV metrics including spectral measures. Third, our study covered only a relatively short time span between stroke and evaluation of outcome. On average, mRS was calculated 6–7 days after stroke, which might only partially reflect long-term outcome after stroke. Fourth, we did not include imaging data into our analysis, which could lend valuable insight into the effect of the lesion site on HR and HRV changes. Fifth, some studies reported an association of blood pressure and HR non-dipping with small vessel disease (SVD) [50]. Higher prevalence of SVD in HR non-dippers may help explain the worse outcome we observed in our cohort. Sixth, we have no data on our patients’ HR dipping-status before stroke. We argued that a blunted HR dip may represent post-stroke ANS dysfunction, but it may well be already present before stroke, possibly indicating higher pre-stroke morbidity. This could also help explain the worse outcome these patients experience. Seventh, even after matching patients for NIHSS and age, the differences between groups remained substantial. However, our cohort was too small to run numerous sub-analyses or adjust for more variables, which limits the generalizability of the results. Eighth, we opted to use outcome data at discharge, because due to the fairly recent establishment of the utilized data warehouse, long-term outcome data is not yet available. Finally, the reported study was a single-center (though multi-site) study, validation of the presented data in other stroke care centers will be necessary.

## Conclusion

Our study showed a distinct pattern of non-dipping HR in patients with short-term unfavorable functional after stroke. This might warrant closer monitoring of HR non-dippers on stroke units and possibly the use of negative chronotropes such as beta-blockers. But whether lowering HR pharmacologically ameliorates stroke outcome is speculative at best and should be thoroughly investigated in further research. HRV measurement in acute stroke patients has proven difficult due to its highly unstable nature that is influenced by many immutable factors in the acute clinical setting. However, a plethora of research has been conducted to simplify its measurement, so there is valid hope that this could lead to increased deployment of this valuable ANS metric in clinical medicine. One could also imagine the inclusion of an automated HR dipping analysis through ECG monitoring on stroke units, alerting clinicians about post-stroke ANS dysfunction.

In conclusion, the availability of large-scale data through data warehousing now allows for a more comprehensive investigation of circadian and other long-term dynamics in cardiac activity and their relationship to stroke outcome. We here report an association between nocturnal non-dipping of HR and unfavorable outcome at discharge in patients with acute stroke. The addition of HR to prediction models based on ML resulted in a significant improvement of prediction performance.

## Supplementary Information

Below is the link to the electronic supplementary material.

Supplementary file1 (DOCX 578 KB)

## Data Availability

The datasets generated and analyzed during the current study are not publicly available due to privacy or ethical restrictions but may be available from the corresponding author on reasonable request. Access to the data will be granted to qualified researchers for purposes of reproducing the results or replicating the procedure.
