# Supplementary material for: Data lake-driven analytics identify nocturnal non-dipping of heart rate as predictor of unfavorable stroke outcome at discharge
Source: J Neurol. 2023 Apr 20;270(8):3810–20. doi: 10.1007/s00415-023-11718-x (PMC10345074; doi:10.1007/s00415-023-11718-x)
Supplement: Supplementary file 1 — Supplementary file1 (DOCX 578 KB) [file 415_2023_11718_MOESM1_ESM.docx]

Data Lake-Driven Analytics Identify Nocturnal Non-Dipping of Heart Rate as Predictor of Unfavorable Stroke Outcome at Discharge

Supplemental Information

Analyses were repeated for ischemic stroke patients only, thus excluding all hemorrhagic stroke patients from the cohort.

**Table S1: Characteristics of patients with good (mRS 0-2) and unfavorable functional outcome (mRS 3-6).** Heart rate variability (HRV) measures are reported as mean over the whole circadian cycle, with exception for the heart rate, which is averaged over the period 22:00-05:00 to capture the difference in circadian rhythms between groups (Figure S1, gray area). *Intravenous thrombolytic therapy (IVT), Endovascular treatment (EVT). **Troponin coding, see Methods.

| **Parameters** | **Total** | **Good** | | **Unfavorable** | **p-value** |
| --- | --- | --- | --- | --- | --- |
|  |  |  |  | |  |
| Number of patients | 248 | 107 | 141 | |  |
| **Epidemiological:** |  |  |  | |  |
| Mean age (years) | 75.7 | 73.6 | 77.3 | | 0.02 |
| Sex (female/male) [%] | 118 / 130  [47.6 / 52.4] | 47 / 60  [43.9 / 56.1] | 71 / 70  [50.4 / 49.6] | | < 0.05 |
| **Clinical:** |  |  |  | |  |
| NIHSS (median) | 5.0 | 5.0 | 6.0 | | < 0.01 |
| Pneumonia [%] | 11 [4.4] | 1 [0.9] | 10 [7.1] | | < 0.05 |
| IVT [%] | 68 [27.4] | 36 [33.6] | 32 [22.7] | | < 0.05 |
| EVT [%] | 34 [13.6] | 22 [18.18] | 17 [10.24] | | 0.05 |
| **Pre-existing conditions:** |  |  |  | |  |
| Diabetes [%] | 73 [29.4] | 27 [25.2] | 46 [32.6] | | 0.21 |
| Hypertension [%] | 209 [84.3] | 82 [76.6] | 127 [90.1] | | < 0.01 |
| Myocardial infarction [%] | 2 [0.8] | 1 [0.8] | 1 [0.7] | | 0.84 |
| Coronary artery disease [%] | 21 [8.5] | 11 [10.3] | 10 [7.1] | | 0.37 |
| **Laboratory values:** |  |  |  | |  |
| Troponin (0/1/2)* [%] | 91/92/65 [36.7/37.1/26.2] | 49/36/22 [45.8/33.6/20.6] | 42/56/43 [29.8/39.7/30.5] | | < 0.01 |
| Mean GFR [ml/min] | 74.0 | 75.1 | 73.2 | | 0.41 |
| **HRV measures (mean):** |  |  |  | |  |
| Heart rate [1/min] | 71.2 | 68.4 | 73.3 | | < 0.01 |
| SDNN [ms] | 47.6 | 45.7 | 49.0 | | 0.44 |
| RMSSD [ms] | 43.1 | 38.7 | 46.4 | | 0.24 |
| LF [ms^2^] | 634.4 | 572.5 | 681.3 | | 0.56 |
| HF [ms^2^] | 1016.1 | 759.4 | 1210.9 | | 0.30 |
| LF/HF [ms^2^] | 2.2 | 2.4 | 2.0 | | 0.08 |


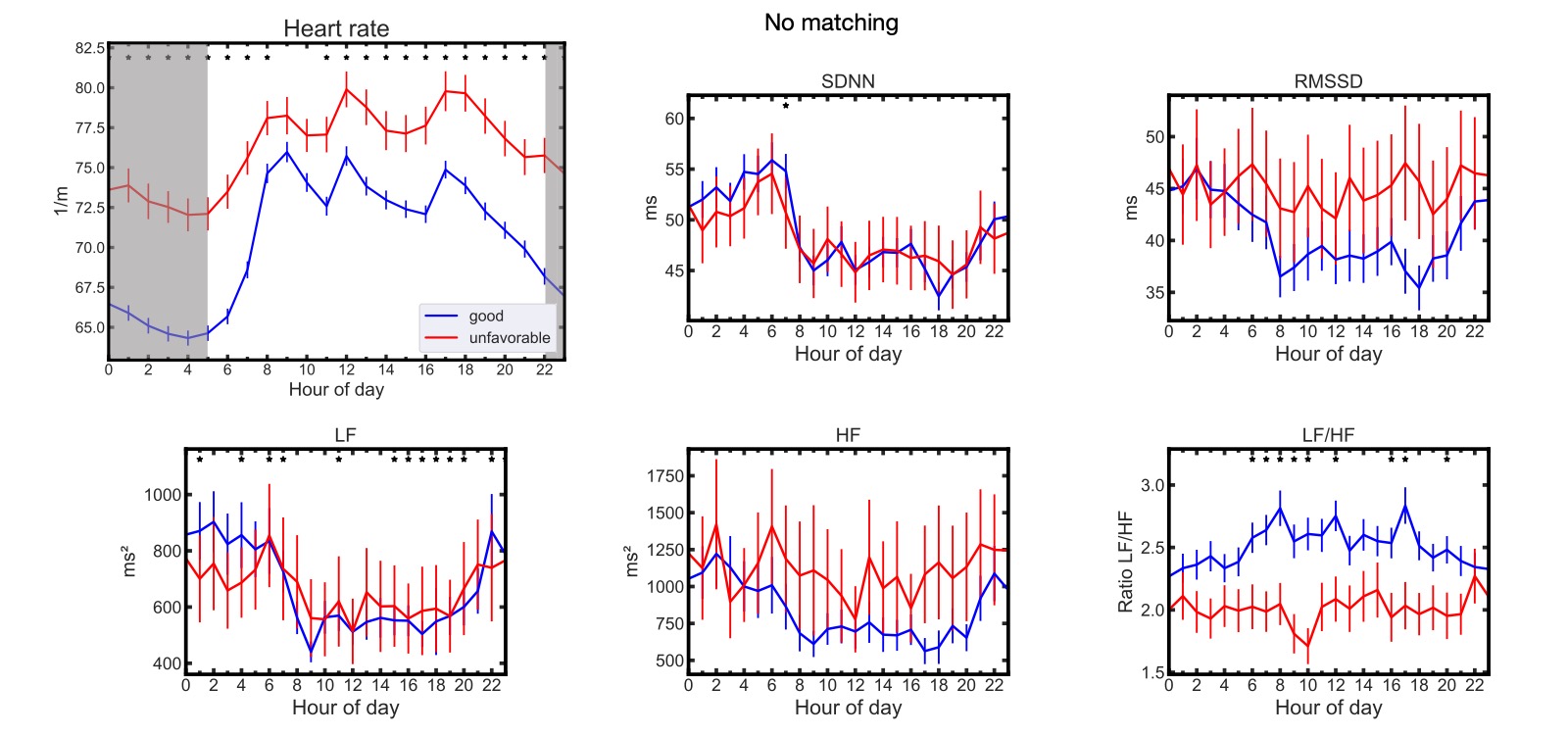


**Figure S1: Circadian dynamics in HR and HRV of ischemic stroke patients with good (blue) vs. unfavorable health state (red) after stroke.** HR values are significantly lower in the good outcome group with a pronounced dip during the night (gray area). Values indicate means with standard deviation*.* * Indicates p < 0.05 for difference between respective individual hours, Mann-Whitney-U-Test, Bonferroni corrected.

**Figure S2*:* Nocturnal HR non-dipping improves stroke outcome prediction as an independent parameter in patients with ischemic stroke.** Top: results for non-matched patients. Bottom: results of patients matched by age and NIHSS. Comparison of prediction performance between feature groups with (orange) and without (blue) utilization of HR (beats per minute, bpm) for prediction and corresponding feature importance for the ML models. For every model type, HR feature importance ranks highly, implying an effective inclusion into the prediction decision. AUC: area under the receiver-operator-characteristics. PSM: Propensity Score Matching. Data displayed as mean with standard deviation. * indicates p < 0.05; ** p < 0.001; standard two-sample location t-test for 50 shuffles of nested cross-validation.
